# Reliability of histopathologic diagnosis of fibrotic interstitial lung disease: an international collaborative standardization project

**DOI:** 10.1186/s12890-021-01522-6

**Published:** 2021-06-01

**Authors:** Robert Camp, Maxwell L. Smith, Brandon T. Larsen, Anja C. Roden, Carol Farver, Andre L. Moreira, Richard Attanoos, Raghavendra Pillappa, Irene Sansano, Alexandre Todorovic Fabro, Robert J. Homer

**Affiliations:** 1 Department of Pathology, Yale University School of Medicine, New Haven, CT 06510 USA; 2 Department of Laboratory Medicine and Pathology, Mayo Clinic, Scottsdale, AZ 85259 USA; 3 Department of Laboratory Medicine and Pathology, Mayo Clinic, Rochester, MN 55902 USA; 4 Department of Pathology, University of Michigan, Ann Arbor, MI 48109 USA; 5 Department of Pathology, New York University School of Medicine, New York, NY 10016 USA; 6 Department of Cellular Pathology, School of Medicine, University Hospital of Wales, Cardiff University, Cardiff, CF14 4XW UK; 7 Department of Pathology, Virginia Commonwealth University School of Medicine, Richmond, VA 23298 USA; 8 Department of Pathology, Vall d’Hebron Hospital, Barcelona, 08035 Spain; 9 Department of Pathology and Legal Medicine, Ribeirão Preto Medical School, University of São Paulo, Ribeirão Preto, São Paulo 14049-900 Brazil; 10 Pathology and Laboratory Medicine Service, VA Connecticut HealthCare System, West Haven, CT 06516 USA

**Keywords:** Interstitial lung disease, Pulmonary fibrosis, Usual interstitial pneumonia

## Abstract

**Background:**

Current interstitial lung disease (ILD) diagnostic guidelines assess criteria across clinical, radiologic and pathologic domains. Significant interobserver variation in histopathologic evaluation has previously been shown but the specific source of these discrepancies is poorly documented. We sought to document specific areas of difficulty and develop improved criteria that would reduce overall interobserver variation.

**Methods:**

Using an internet-based approach, we reviewed selected images of specific diagnostic features of ILD histopathology and whole slide images of fibrotic ILD. After an initial round of review, we confirmed the presence of interobserver variation among our group. We then developed refined criteria and reviewed a second set of cases.

**Results:**

The initial round reproduced the existing literature on interobserver variation in diagnosis of ILD. Cases which were pre-selected as inconsistent with usual interstitial pneumonia/idiopathic pulmonary fibrosis (UIP/IPF) were confirmed as such by multi-observer review. Cases which were thought to be in the spectrum of chronic fibrotic ILD for which UIP/IPF were in the differential showed marked variation in nearly all aspects of ILD evaluation including extent of inflammation and extent and pattern of fibrosis. A proposed set of more explicit criteria had only modest effects on this outcome. While we were only modestly successful in reducing interobserver variation, we did identify specific reasons that current histopathologic criteria of fibrotic ILD are not well defined in practice.

**Conclusions:**

Any additional classification scheme must address interobserver variation in histopathologic diagnosis of fibrotic ILD order to remain clinically relevant. Improvements to tissue-based diagnostics may require substantial resources such as larger datasets or novel technologies to improve reproducibility. Benchmarks should be established for expected outcomes among clinically defined subgroups as a quality metric.

**Supplementary Information:**

The online version contains supplementary material available at 10.1186/s12890-021-01522-6.

## Background

Interstitial lung disease (ILD) refers to a range of diagnostic entities which show varying degrees of inflammation and fibrosis [1]. Idiopathic pulmonary fibrosis (IPF) is the most common idiopathic ILD and the most lethal, with 50 % mortality of patients within 3–5 years after diagnosis [2]. IPF was initially considered a chronic inflammatory disease and was therefore commonly treated with immunosuppression. More recently, it has been shown that immunosuppressive therapies have a detrimental effect while antifibrotic drugs are effective in slowing the progression of the disease [3, 4]. Other ILDs such as collagen vascular disease associated ILD and fibrotic hypersensitivity pneumonitis (HP) are still potentially treated with immunosuppression even though so called progressive fibrotic ILD of any association may ultimately benefit from anti-fibrotics [5]. It is therefore essential that a distinction be made between IPF and these other ILDs.

Current IPF diagnostic guidelines assess criteria across clinical, radiologic and pathologic domains which are then combined to create a probabilistic estimate of the diagnosis [6–8]. Modern consensus histopathologic criteria for evaluation of IPF include (1) extent and pattern of fibrosis, (2) extent and pattern of inflammation and (3) presence of other features (e.g. foreign material) that would indicate another diagnosis. An underlying principle of this approach is that the clinical entity of IPF is uniquely associated with the histologic pattern of usual interstitial pneumonia (UIP) which is distinguishable from other patterns in other diseases. However, the utility of these histologic criteria is limited, because of significant interobserver variation [9–12]. We were interested in understanding the underlying basis for this variation as that has not been well described in previous studies. We further hypothesized that understanding this variation would allow us to improve performance by developing improved criteria and testing it on a subset of previously evaluated cases and on a set of new cases.

## Materials and methods

A website was created for this project which displayed both fixed images of selected features relevant to diagnosis of IPF and whole slide images (WSI) of cases of ILD [13]. These images were displayed adjacent to questions concerning presence or absence of specific features and, in cases of WSI, final diagnosis (Table 1, Additional file 2: Figs. S1–S2A–B) (See reference [13] for web address). In those ILD cases which involved multiple slides, selected representative slides were chosen with a range of 1–4 WSI per case. The website collected user specific answers based on unique sign ins. The website is publicly accessible and currently displays all of the images used in this study and a summary of the data generated. No identifiable patient information is available on that website or was used in this project. The pathologists who participated in this study are all senior academic thoracic pathologists, many of whom have published on this and related topics and have recognized expertise by serving as referral specialists in tertiary academic centers [1, 14].

**Table 1:** Questions used for criteria sets and whole slide images

Fibrosis criteria set	Is the fibrosis severe (yes, no, uncertain)? Is the pattern of fibrosis patchy, diffuse or honeycombing only? If the fibrosis is patchy, is the distribution subpleural/paraseptal, airway centered or uncertain?
Inflammation criteria set	Is there dense inflammation away from scar (yes, no, uncertain)?
Fibroblast foci criteria set	Are fibroblast foci adjacent to scar at the boundary of normal and fibrotic lung (readily identified, rare, none, cannot determine)?
Granuloma criteria set	Does inflammation include granulomas (well formed, poorly formed, scattered giant cells only, none of these, or uncertain)?
Whole slide images, first round only	Is the fibrosis severe (yes, no)? Is the pattern patchy, diffuse or honeycombing only? Is the distribution of fibrosis subpleural/paraseptal, airway centered or uncertain/mixed?
Whole slide images, final round only	Is the fibrosis severe (yes, no, honeycombing only) Is the distribution of fibrosis subpleural/ paraseptal, irregular, airway centered, diffuse or uncertain?
Whole slide images, both rounds	Are fibroblast foci readily identified, rare, none or cannot be determined? Are there non-UIP features present (dense inflammation away from scar, granuloma, organizing pneumonia, smoking related interstitial fibrosis or other)? Is this definite, probable, possible^a^ or not UIP/IPF?

^a^Several of the terms we used are not identical to that used in the ATS guidelines. For extent of fibrosis, instead of “is the fibrosis severe”, the 2011 ATS requires “Evidence of marked fibrosis/architectural distortion, +/− honeycombing” while the 2018 ATS requires “Dense fibrosis with architectural distortion (i.e., destructive scarring and/or honeycombing)”. For extent of inflammation, instead of “Is there dense inflammation away from scar” the 2011 ATS requires “marked interstitial inflammatory cell infiltrate away from honeycombing” while the 2018 ATS requires “areas of interstitial inflammation lacking associated fibrosis” (2018). We used the 2011 nomenclature of “possible” UIP instead of the 2018 ATS and Fleishner nomenclature of “indeterminate” for UIP. We do not believe that the meaning of any the phrases we used are substantially different from those of the various guidelines and were understood as such by the participants

This study consisted of two rounds of review. In the first round, pathologists were asked to (1) categorize fixed images of lung using standard criteria used for diagnosis of ILD and (2) to categorize cases of ILD using whole slide images. The criteria sets were created for the domains of fibrosis (25 images), inflammation (9 images), granulomas (10 images) and fibroblast foci (7 images). These images were selected by the senior author to include a range of characteristics required to make a diagnosis of UIP and other ILD diagnoses. Eight pathologists provided answers to those images. The whole slide images were created from thirty wedge biopsy cases and included a range of ILD diagnoses. These cases were selected by the senior author to include the range of cases seen in routine practice. Seven pathologists provided answers for these cases.

After the initial round, the data was evaluated and shared with the participants. Multiple conference calls were made among the authors to discuss cases and criteria with discrepancies. A consensus document was circulated among the authors for evaluation and a final version was used for a second round of WSI cases. For the second evaluation round, the senior author selected twenty cases (ten WSI cases from the first round and ten new cases from two of the participants’ routine sign out) for review. Ten pathologists provided answers for those cases. The criteria assessments were not repeated.

Spearman’s rank correlation analysis was performed using Prism v 8.4.3 for MacOS.

## Results

### Initial evaluation round

In the first round, participants reviewed a set of fixed images (“criteria sets”) from the project website which were designed to clarify the use of specific criteria relevant to fibrotic ILD as well as WSI (Table 1) [13]. Criteria sets were created for the domains of fibrosis (25 images), inflammation (9 images), granulomas (10 images) and fibroblast foci (7 images). Eight pathologists participated in the criteria set portion of the survey. Consensus, as defined by agreement among six or more of eight pathologists, varied among criteria relevant to a diagnosis of UIP (Additional file 1; Additional file 2: Figs. S3–S5). For example, the pattern and extent of fibrosis typically achieved consensus from 68 to 76% of the time (Table 2, Additional file 2: Fig. S3a–c) depending on exactly how this is evaluated. There was less agreement for questions aimed at extent and type of inflammation, including evaluation of dense inflammation away from scar and the distinction between well or poorly formed granuloma vs. scattered giant cells (Table 2, Additional file 2: Figs. S4, S5).

**Table 2:** Interobserver concordance on various criteria set images

	Number of images with consensus
Severe versus not severe fibrosis or uncertain	18/25 (72 %)
Honeycombing or patchy fibrosis versus diffuse fibrosis	17/25 (68 %)
Presence of honeycombing or subpleural/ paraseptal fibrosis versus other pattern	19/25 (76 %)
Presence of none or rare fibroblast foci versus readily identified fibroblast foci	5/7 (71 %)
Presence of well or poorly formed granulomas versus scattered giant cells or no granuloma	7/10 (70 %)
Dense inflammation away from scar	4/9 (44 %)
Well-formed or poorly formed granuloma vs. scattered giant cells	4/7 (57 %)

**Fig. 1:**
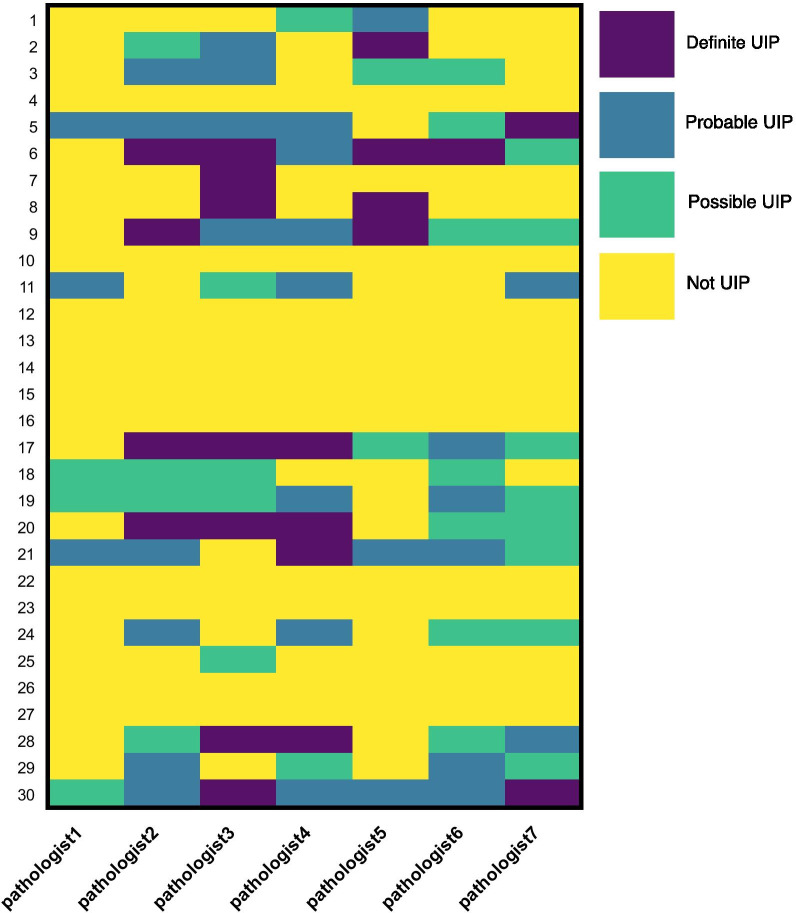
Variation in overall diagnosis of cases in initial round including definite UIP, probable UIP, possible UIP and not UIP. WSI case numbers are listed on the left. Specific pathologists are listed along the bottom

Thirty wedge biopsy cases for evaluation of ILD were also selected by the senior author for the project website including two thought most likely UIP, fourteen thought most likely not UIP and fourteen that were thought ambiguous [13]. Seven pathologists answered nearly all of the WSI cases (Fig. 1, Additional file 1). In order to analyze the data, we grouped together definite and probable UIP vs. possible and not UIP. We considered consensus to be agreement among at least five of seven pathologists. Four cases were identified as probable or definite UIP pattern including the two initially thought to be UIP (cases 5, 6, 21, 30). Fourteen cases were nearly universally thought not, or at most possible, UIP, all of which were among the cases initially not thought to be UIP (cases 4, 7, 10, 12–16, 18, 22, 23, 25–27). Thirteen of those fourteen had areas of hyaline membranes, extensive organizing pneumonitis, marked increase in eosinophils, diffuse hyalinized fibrosis (“smoking related interstitial fibrosis”) and / or irregular fibrosis (see below for definition) with patchy lymphoid infiltrate and/or granulomas. The remaining twelve cases had variable interpretation by the participants with only seven of these cases reaching consensus. Even in cases ultimately reaching consensus, there was marked variation in interpretation across all criteria (Additional file 1). For example, while case eight met consensus, only four pathologists thought that it showed severe fibrosis, and two of those thought distribution was either airway centered or diffuse rather than patchy and paraseptal /subpleural. Those who thought fibrosis was not severe thought distribution was either uncertain or airway centered. Two thought there was excess inflammation. The performance of pathologists in the initial phase of this project was consistent with the existing literature and reinforces the concept that pathologist confidence is an important component of ILD diagnosis [15].

### Discussion phase

**Fig. 2:**
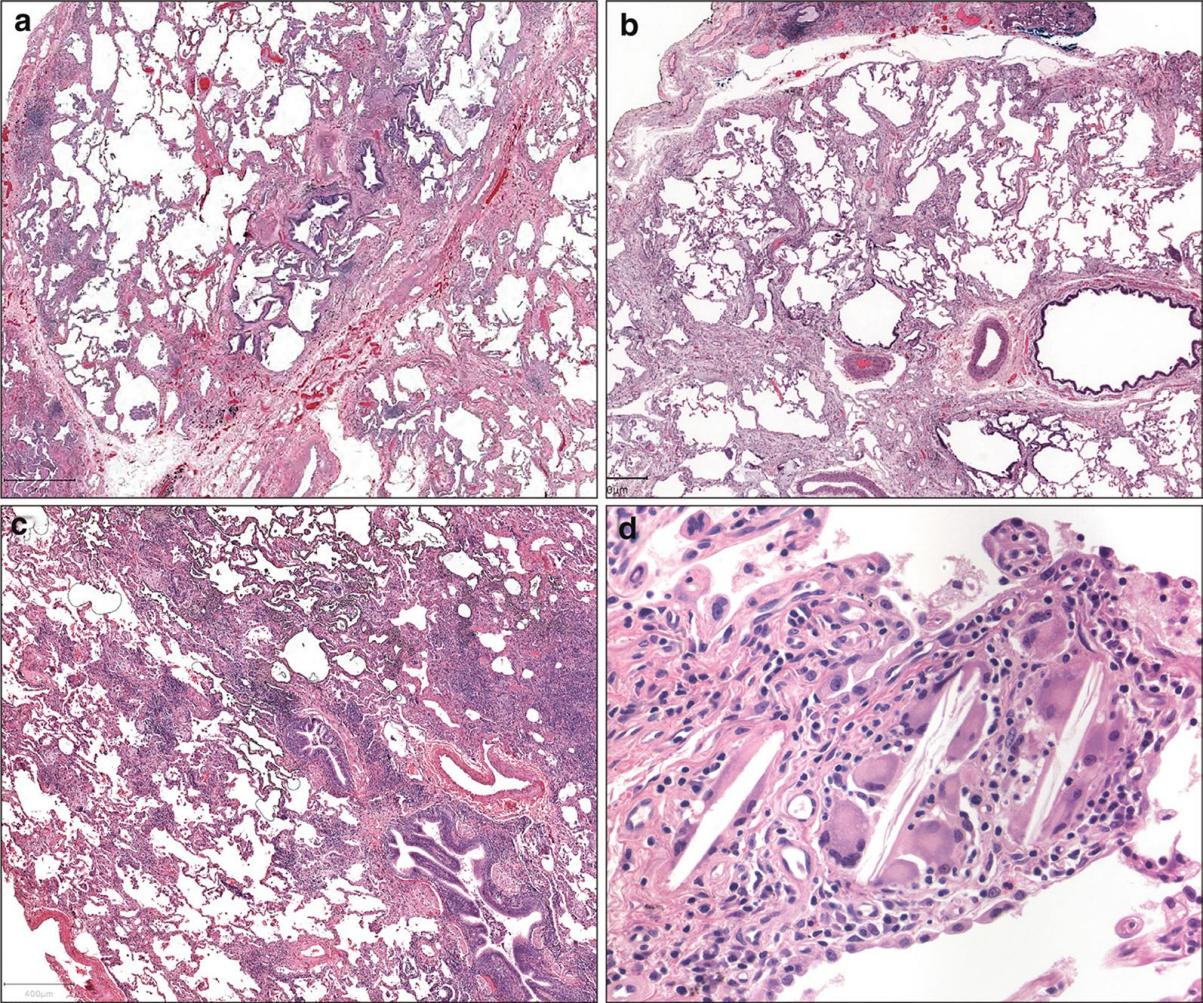
Selected criteria set images taken from the project website illustrative of problems and potential solutions in diagnoses of chronic fibrotic ILD [13]. **a** Fibrosis criteria set image 5, also used as Inflammation criterion set image 4, **b** Fibrosis criteria set image 25, **c** Fibrosis criteria set image 2, **d** granuloma criteria image 1. Figure 2a shows fibrosis which is focally severe predominantly around the airways in the center of the image although there is a connection to the adjacent interlobular septa. Figure 2b shows mildly cellular fibrosis without dense (“collagen”) fibrosis. The fibrosis seems to merge continuously into the non-fibrotic region without an easily drawn boundary. There is marked bronchiolectasis (the airway in the right middle is massively enlarged relative to the adjacent artery) while there is minimal fibrosis around that airway itself. This pattern of fibrosis is not clearly defined in established criteria and generates conflicting interpretation in the diagnostic categories. See text for additional discussion

We then reviewed a number of the most problematic of the criteria set images from the project website in an effort to derive more specific criteria for severity of fibrosis, distribution of fibrosis, extent of inflammation and nature of granulomas (Fig. 2a–d). We attempted to create these rules by consensus to determine if they would reduce interobserver variability. While these rules were created independent of any attempt to determine if those were clinically predictive of outcome, the criteria developed largely paralleled the clinical practice of the participants. For severe fibrosis, we adopted two independent criteria. (1) We considered that a case displayed severe fibrosis if at least 25% of the slide showed established fibrosis and the fibrotic process was distributed across the entire slide even if that process was patchy. (2) While bronchiolectasis can be seen in honeycombing, we considered that bronchiolectasis reflected severe fibrosis even in presence of much milder fibrosis since that pattern has been associated with radiologic honeycombing, possibly due to severe fibrosis not seen in the plane of histologic Sect. [16]. On the other hand, while honeycombing is severe fibrosis by definition, we considered honeycombing in lung tips only as non-specific. A review of Fig. 2 shows how this worked in practice. The amount of fibrosis in Fig. 2a is less than 25%, changes are not continuous across the entire slide and there is no honeycombing, so it would fail to meet criteria for severe fibrosis. For Fig. 2b, on the other hand, the presence of bronchiolectasis on a background of patchy mild to moderate fibrosis would support the final interpretation of this image as patchy severe fibrosis.

Among fibrosis criteria set images, there was wide variation in evaluation of distribution of fibrosis (Additional file 2: Fig. S3c). We had difficulty categorizing the distribution of fibrosis in some cases since we considered diffuse fibrosis to show expansion of septa without residual normal septa while patchy fibrosis should show severe established fibrosis adjacent to relatively normal lung. However, in some cases (Fig. 2c, which was used as fibrotic criteria set image 2), there is neither uniform involvement nor a strong boundary between normal and fibrotic lung. We proposed creation of a new category we called irregular fibrosis which shows patchy expansion (“thickening”) of alveolar septa with some residual normal alveoli. Some might consider irregular fibrosis a milder form of either diffuse or patchy fibrosis. It would not support a diagnosis of UIP. We thought airway-based fibrosis is only confidently diagnosed when it is either an isolated or strongly dominant finding. While Fig. 2a shows an airway with fibrosis, that process shows bridging to an area of subpleural/paraseptal fibrosis and we therefore did not consider that to represent airway centric fibrosis. Similarly, we considered that honeycombing around airways is also not airway centered fibrosis if it is connected to the periphery. None of the cases in our study had examples of airway-to-airway fibrosis which some have proposed to represent airway centric fibrosis [17] nor did any of our participants consider this a common finding in their practice.

Inflammation criteria set images with marked disagreement was seen in cases with isolated clusters of lymphoid cells, commonly associated with some degree of fibrosis (Fig. 2a). The conventional criteria require inflammation to be away from scar to be considered significant, but we agreed that too much inflammation in areas of only mild fibrosis would also be inconsistent with UIP. However, there was little agreement on how much of either was acceptable or required for that to be true.

Among granuloma criteria set images, there was a striking disagreement on interpretation of cholesterol clefts in the giant cells (Fig. 2d). The presence of cholesterol clefts in scattered macrophages in airspaces may reflect response to degenerating/ necrotic material and is typically thought to be of no significance. However, we ultimately recognized that true granulomas, especially those present within the interstitium, might also have such clefts and can be recognized as such if the architecture and background cellular composition are otherwise typical.

### Second (final) evaluation round

**Fig. 3:**
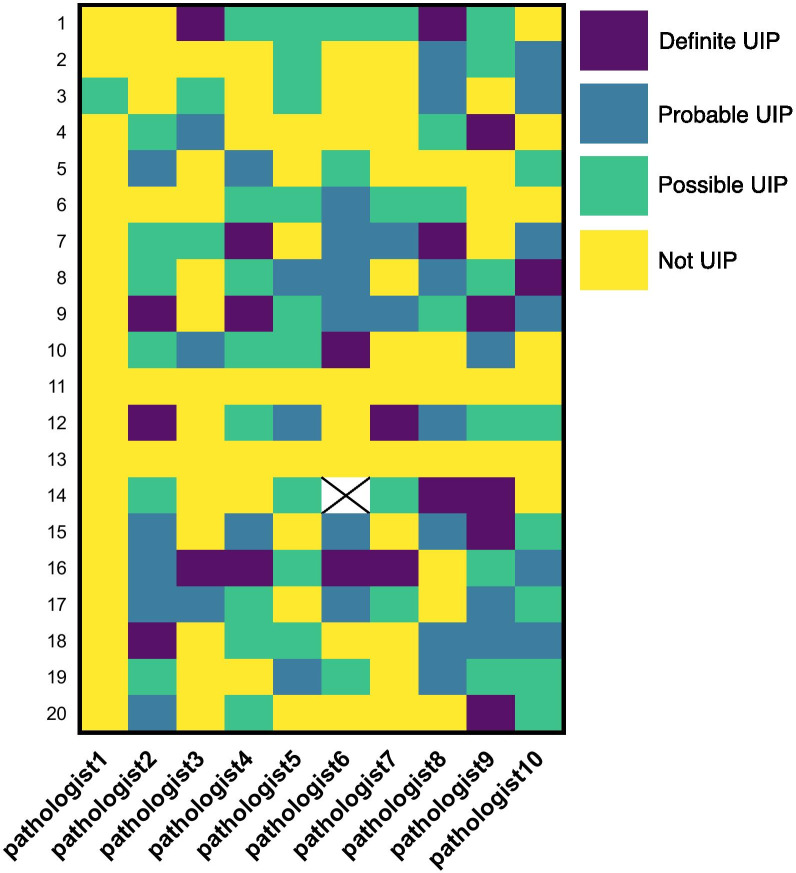
Variation in overall diagnosis of cases in final round including definite UIP, probable UIP, possible UIP and not UIP. WSI case numbers are listed on the left. Cases 1–29 are taken from the initial round while cases 31–40 are new. Specific pathologists are listed along the bottom. Case 34 was not diagnosed by pathologist 6

For a second and final evaluation round, we selected ten WSI cases from the first round as well as ten new cases from two of the participants’ routine sign out practice. Nine of the ten selected cases from the original set were originally considered ambiguous by the senior author (other than case 18). In the second group of ten, four were confidently considered by the senior author as not UIP (case 31, 33, 34, 40, Fig. 3) and the remaining six were considered ambiguous. Ten pathologists evaluated all twenty cases. We did not repeat the criteria assessments. The cases were reviewed six months after the initial round, at which point we assumed that the participants would not recall their initial impression of the cases. Questions were similar to those used in the first round, but the first three questions were consolidated into two (Table 1; Additional file 2: Fig. S2b).

We again grouped together definite and probable UIP vs. possible and not UIP. Since ten pathologists participated in this round, we considered consensus to be agreement among at least seven of the pathologists. Using the revised diagnostic criteria, twelve of the twenty cases reached consensus (Cases 1–6, 10–11, 13–14, 20, Fig. 3, Additional file 1). The specific new category of irregular fibrosis was used in eleven cases. It did not increase reproducibility nor was it used to consistently to rule in or rule out IPF/UIP. As in the first round, a confident diagnosis of possible or non-UIP was highly reproducible with all five cases (one from initial set and four from second set) initially thought to not be UIP by the senior author achieving consensus.

**Fig. 4:**
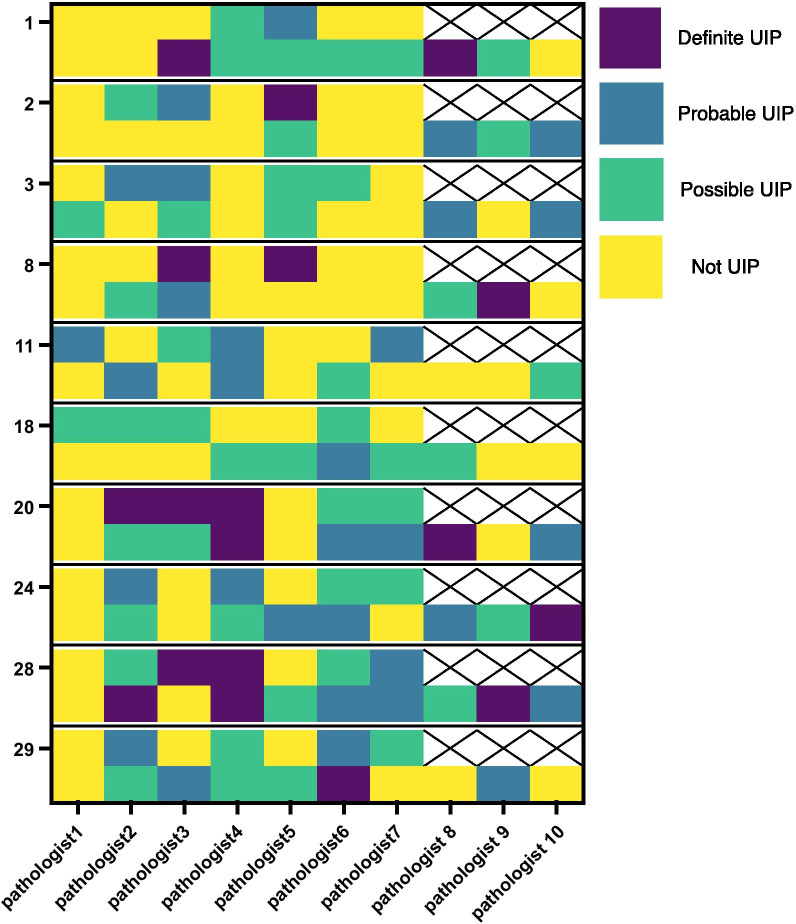
Comparison of diagnoses for WSI cases evaluated in both rounds including definite UIP, probable UIP, possible UIP and not UIP. Two sets of diagnoses are listed for each case. The upper set of diagnoses are from first round and lower are from second. WSI case numbers are from the first round. Specific pathologists are listed along the bottom. Pathologists 8, 9, and 10 did not make diagnoses in the first round

If we restrict analysis to the ten cases seen in both rounds, six achieved consensus in both rounds, two cases did not achieve consensus in either round (cases 20 and 28, Fig. 4), one case lost consensus (case 11, Fig. 4.) and one case gained it (case 24, Fig. 4). If we restrict analysis only to those pathologists who evaluated these ten cases in both rounds, two cases (cases 20 and 24, Fig. 4) improved agreement with one case now achieving consensus while the rest remained essentially the same (zero or one changed diagnoses) (Fig. 4).

We were interested to know whether the variability in round one and two could be due to specific pathologists who had consistent differences of opinion from the rest since the rate of diagnosis of UIP varied markedly with the pathologist e.g. in the first round one pathologist diagnosed definite or probable UIP three times, while another two diagnosed UIP eleven times (Fig. 1). There was a positive correlation between rate of diagnosis by pathologist of definite or probably UIP in first and final round with a Spearman’s r value of 0.49 although with a p value of 0.27. On the other hand, we do note that two of the three pathologists with the lowest rate of UIP diagnosis in the first round remained in the bottom three in the second round suggesting that this may play a role for some pathologists.

## Discussion

All three of the recent standard criteria for idiopathic pulmonary fibrosis and the new hypersensitivity pneumonitis criteria include histologic criteria [6–8, 18]. However, there are no large validated series of images or cases derived from daily practice that serve as reference standards. Possibly as a result, significant interobserver variation exists limiting the utility of this approach [9–12, 19]. Notably all of the criteria use various quantitative assessments that are not given more specific definitions, nor are rules provided when criteria conflict. For example, lymphoid aggregates have long been noted in conventional histology of IPF and have been documented in more recent molecular characterization [19–24]. On the other hand, excess inflammation away from fibrosis is still considered to argue against a UIP diagnosis by raising consideration for HP, other hypersensitivity reaction or occult collagen vascular disease. Perhaps not surprisingly, therefore, and similar to our work, one previous study limited to IPF showed that excess inflammation and/or presence of giant cells were areas of diagnostic difficulty [9]. In comparison to that study, we examined a broader range of diagnoses and involved a larger number of pathologists. The difficulties in this differential have been explored in more detail recently by some of us [14]. Our data here documents that many of the concerns raised in that article are problems in actual practice. On the other hand, we did find that cases with findings that are considered to be inconsistent with UIP such as smoking related fibrosis, some cases with patchy inflammation and/or granulomas and cases with acute lung injury are readily distinguished from UIP, even in a whole slide imaging format. In general, we suspect that cases that have high confidence that they are not UIP are strongly reproducible as such, although we have not formally tested that.

It is important for pathologists to appreciate that there is significant mortality associated with wedge lung biopsy [25, 26]. We also note that, possibly as a result, one current trend in ILD diagnostics is to discard specific histologic categories in favor of a more general progressive fibrotic phenotype [27]. It may also be that there are no fixed borders among fibrotic ILD and that all such efforts at distinction may fail due to lack of underlying discrete categories [28].

The improvement we identified with our revised criteria was modest at best. While the criteria we used were somewhat arbitrary, they reflect the consensus of a group of pathologists who are extremely active in the field. Notably, the existing criteria have also never been subject to clinical validation but were only generated by consensus. Thus our approach is not different from that which is standard in the field. Our approach was also not too dissimilar to that of the commonly used Delphi system of developing expert consensus when data is lacking. Finally, our goal was primarily to determine if improved criteria could be developed rather than prove that that system improved clinical prediction. We did not attempt to determine who was “right” in this study e.g. by comparison to outcomes, for that reason. We note that if the pathologic criteria are not reproducible, they cannot be tested either by comparison to clinical features or in clinical trials. As a result, we have not considered location or number of biopsies, nor radiologic impression in understanding our data. While those are important to consider in making a multidisciplinary diagnosis, they do not explain the problems we have outlined here. It is also common to assess interobserver variation by use of various statistical tool e.g. Fleiss kappa. However, we note that the extent of disagreement will be heavily influenced by the case mix of patients selected for biopsy. We have not selected sequential cases from our institutions for that reason. While consensus was achieved even in the difficult cases, it is not standard of care for cases to be reviewed by a large panel such as this. The way in which we grouped cases for consensus may also have varying clinical consequences depending on other (clinical and radiologic) factors. Consequently, there will be a significant number of cases for which the consensus opinion will not be the one used clinically. One interpretation therefore is that there is a group of biopsies for which consensus may not be reached even with more precise criteria but that the number of those will vary from institution to institution. One significant limitation of our data is that we did not identify and then systematically re-analyze a large number of discordant cases. We think this is worth exploring going forward.

It is possible that intrinsic propensity among pathologists for diagnosing UIP/IPF accounts for some of these discrepancies. We therefore suggest that benchmarking rates of ILD diagnosis among pathologists needs to be further explored to understand this source of diagnostic variation. Other areas of pathology e.g. evaluation of Barrett’s esophagus, have successfully adopted this concept using web based approaches [29]. Finally, it may be necessary to combine this kind of analysis with newer technologies including image analysis and biomarkers to create the desired result. Such technologies are in development but are not yet routinely incorporated into clinical practice [30].

## Supplementary Information

**Additional file 1**. Complete survey answers. An excel file containing all responses by each participant for each image and case in both rounds of evaluation.

**Additional file 2**. Additional figures including screen shots of the website and graphic representation of responses to various specific questions. **Fig. S1**. A screen shot from the project website illustrating one of the fibrosis criteria set images with adjacent questions [13]. Above the histologic image are a series of boxed numbers. Selecting one of those boxes will select the corresponding image. The upper left-hand corner has arrows which move the viewer though the cases one at a time. The box in that corner moves the viewer back to the home page. The questions are listed on the left. In this particular example, since honeycombing is selected, the user is not asked to determine a distribution. If patchy or diffuse was selected, then that option would be available. **Fig. S2**. A screen shot from the project website illustrating one of the WSI with adjacent questions [13]. (A) Screen shot from the initial round. Above the histologic image are a series of four clickable slides. This shows that the case has four slides and allows navigation within the case. The upper left-hand corner has arrows which move the viewer though the slides one at a time. At the last slide, the arrow will take the viewer to the next case. The box in that corner moves the viewer back to the home page. The questions are listed on the left. Answers to questions persist across the entire case. The lower left-hand corner has vertical arrows above and below the magnification number. Magnification can be changed by clicking those arrows or by scrolling up or down within the image. The red and white pencils were designed to be used for pointing/ circling various features but were not used by participants. (B) Screen shot from the second round. This is similar to 2 A, but the first three questions have been consolidated to two. **Fig. S3**. Variation in evaluation of fibrosis criteria by image by pathologist. In all three figures, the left-hand side lists each image number. Specific pathologists are listed along the bottom. (A) Severity of fibrosis (severe, not severe, uncertain) (B) Pattern of fibrosis (diffuse, honeycomb only or patchy) (C) Distribution and severity of fibrosis (honeycomb, diffuse, subpleural/paraseptal, uncertain, airway centered). **Fig. S4**. Variation in evaluation of presence of dense inflammation away from scar (yes, no, uncertain) by image by pathologist. The left-hand side lists image number. Specific pathologists are listed along the bottom. **Fig. S5**. Variation in evaluation of granuloma criteria (well-formed granuloma, poorly formed granuloma, scattered giant cells only, none, uncertain) by image. The left-hand side lists image number. Specific pathologists are listed along the bottom.

## Data Availability

All images on which this paper is based can be accessed at the website referenced in the paper. All data from the results of this study is included in the additional material as Additional file 1.

## Abbreviations

UIP: Usual interstitial pneumonia
ILD: Interstitial lung disease
HP: Chronic hypersensitivity pneumonitis
WSI: Whole slide imaging
