# Supplementary material for: Reliability of histopathologic diagnosis of fibrotic interstitial lung disease: an international collaborative standardization project
Source: BMC Pulm Med. 2021 Jun 1;21:184. doi: 10.1186/s12890-021-01522-6 (PMC8170950; doi:10.1186/s12890-021-01522-6)
Supplement: Supplementary file 2 — Additional file 2. Additional figures including screen shots of the website and graphic representation of responses to various specific questions. Fig. S1. A screen shot from the project website illustrating one of the fibrosis criteria set images with adjacent questions [13]. Above the histologic image are a series of boxed numbers. Selecting one of those boxes will select the corresponding image. The upper left-hand corner has arrows which move the viewer though the cases one at a time. The box in that corner moves the viewer back to the home page. The questions are listed on the left. In this particular example, since honeycombing is selected, the user is not asked to determine a distribution. If patchy or diffuse was selected, then that option would be available. Fig. S2. A screen shot from the project website illustrating one of the WSI with adjacent questions [13]. (A) Screen shot from the initial round. Above the histologic image are a series of four clickable slides. This shows that the case has four slides and allows navigation within the case. The upper left-hand corner has arrows which move the viewer though the slides one at a time. At the last slide, the arrow will take the viewer to the next case. The box in that corner moves the viewer back to the home page. The questions are listed on the left. Answers to questions persist across the entire case. The lower left-hand corner has vertical arrows above and below the magnification number. Magnification can be changed by clicking those arrows or by scrolling up or down within the image. The red and white pencils were designed to be used for pointing/ circling various features but were not used by participants. (B) Screen shot from the second round. This is similar to 2 A, but the first three questions have been consolidated to two. Fig. S3. Variation in evaluation of fibrosis criteria by image by pathologist. In all three figures, the left-hand side lists each image number. Specific pathologists are listed [file 12890_2021_1522_MOESM2_ESM.pdf]

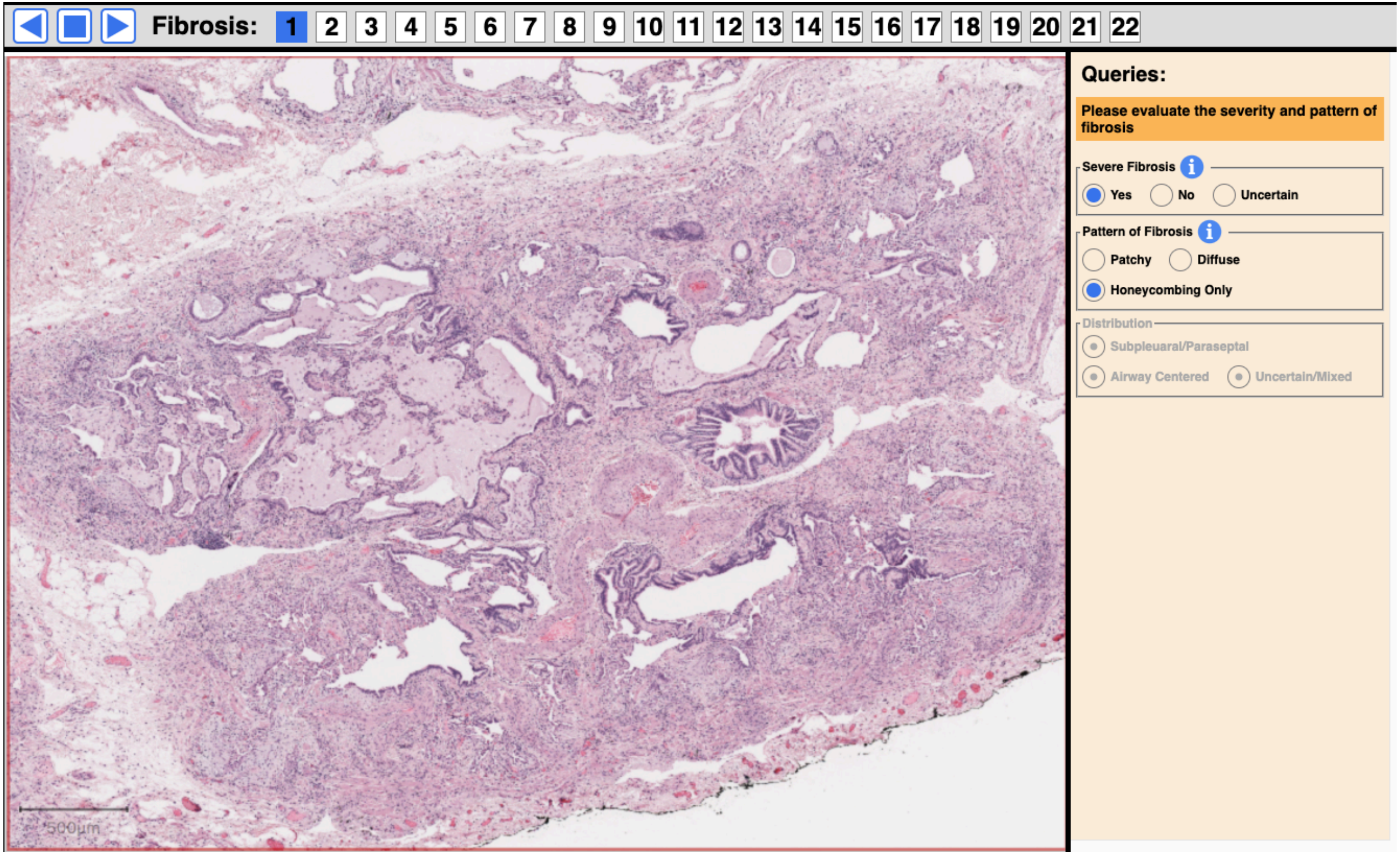

Supplemental figure 1

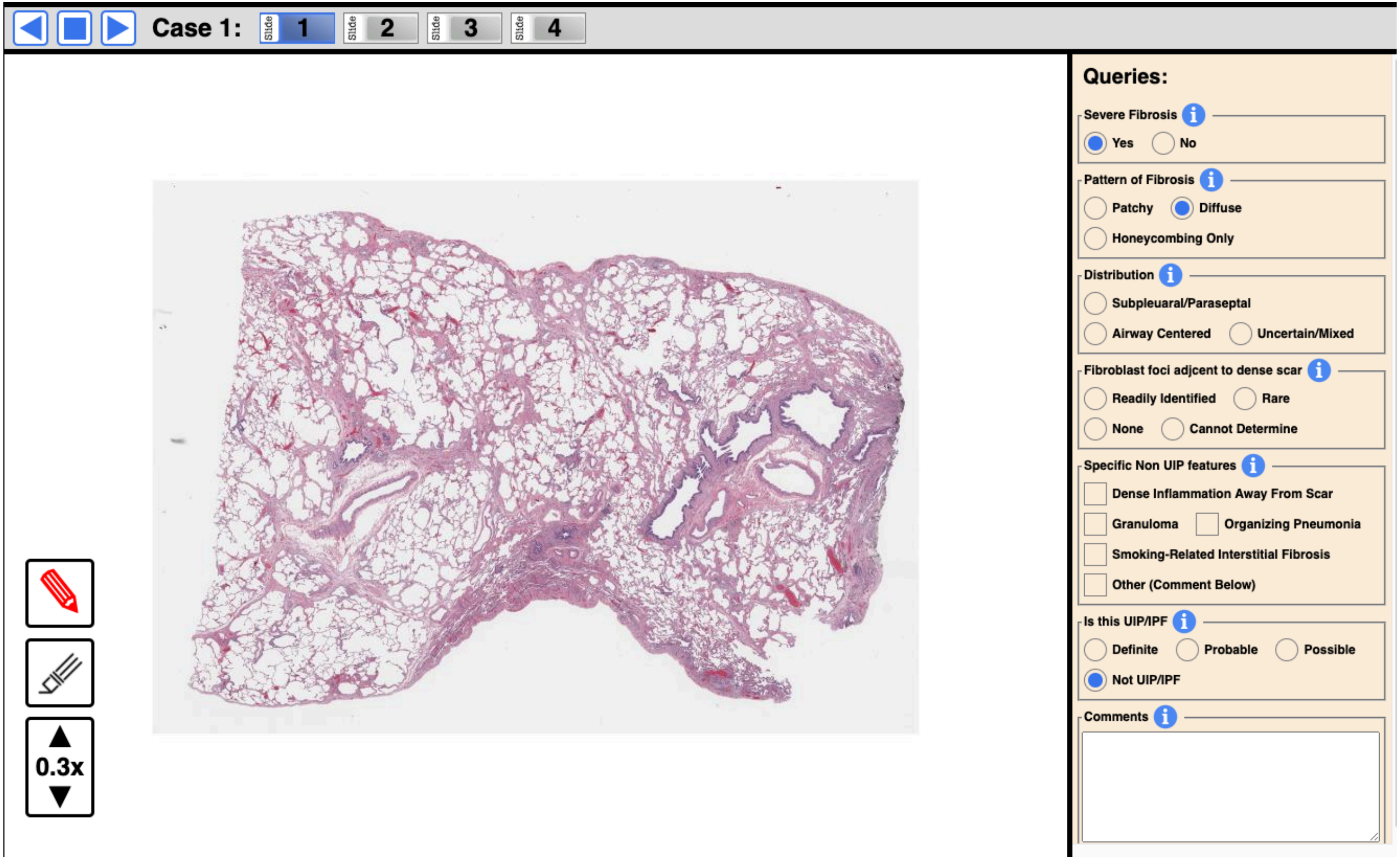

Supplemental figure 2a

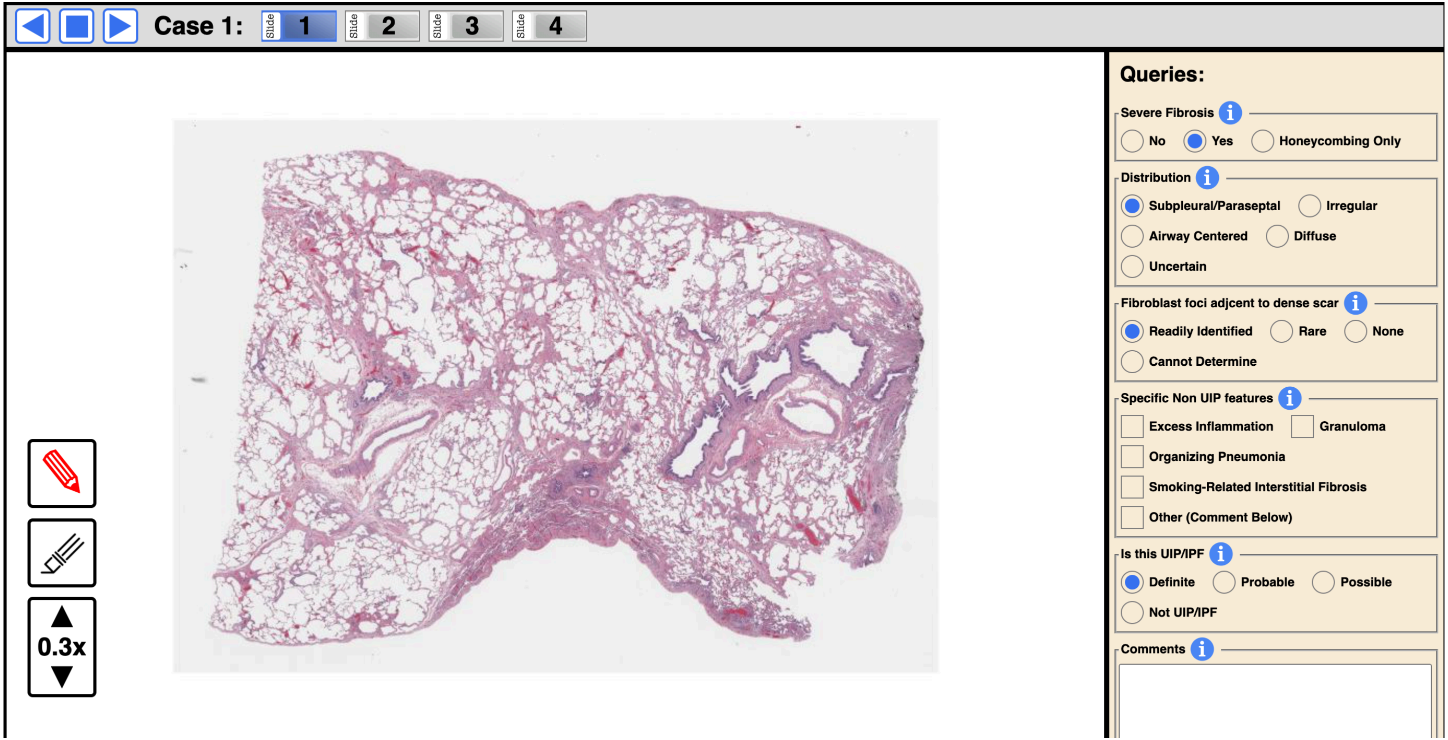

Supplemental figure 2b

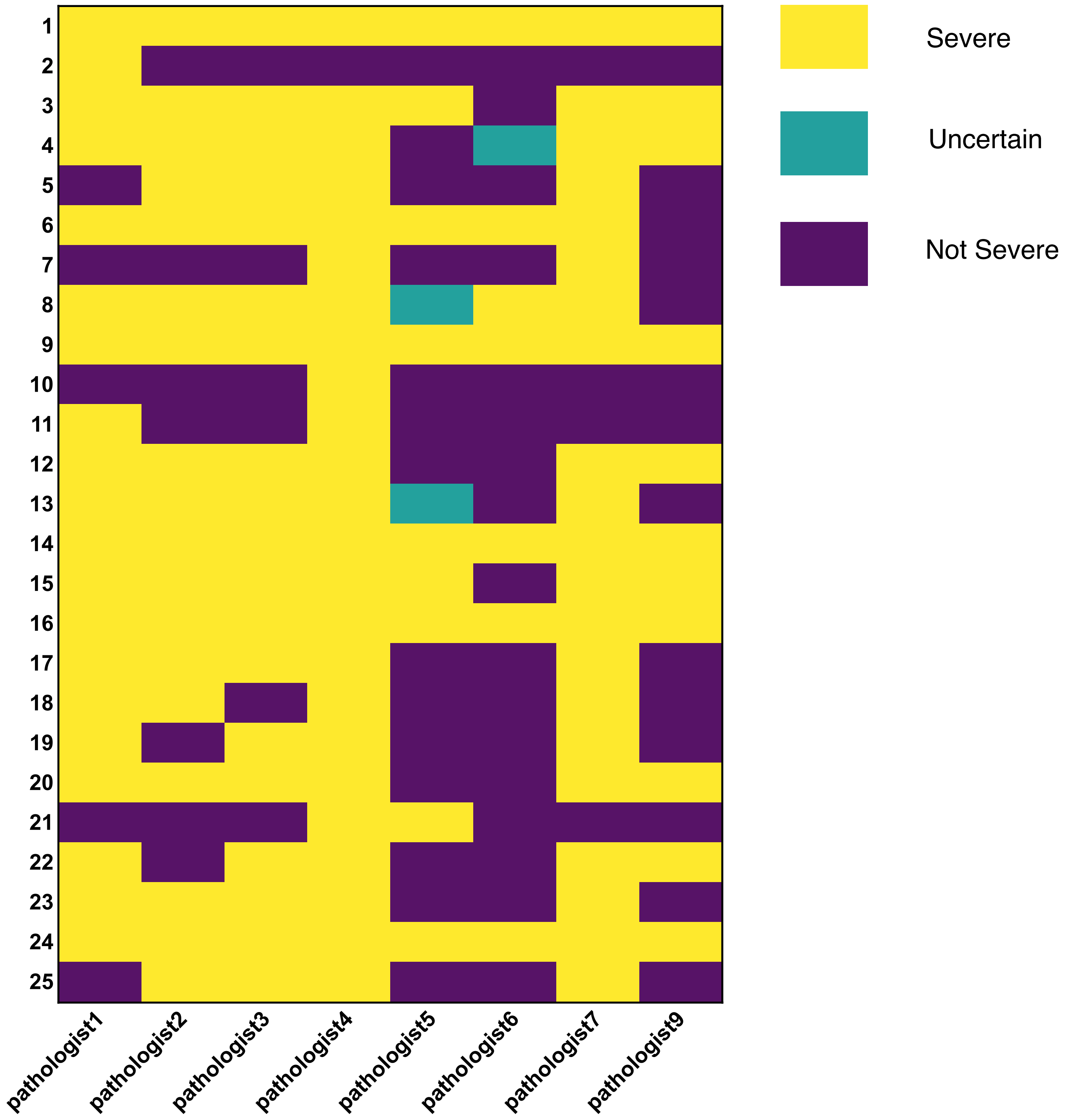

Supplemental figure 3a

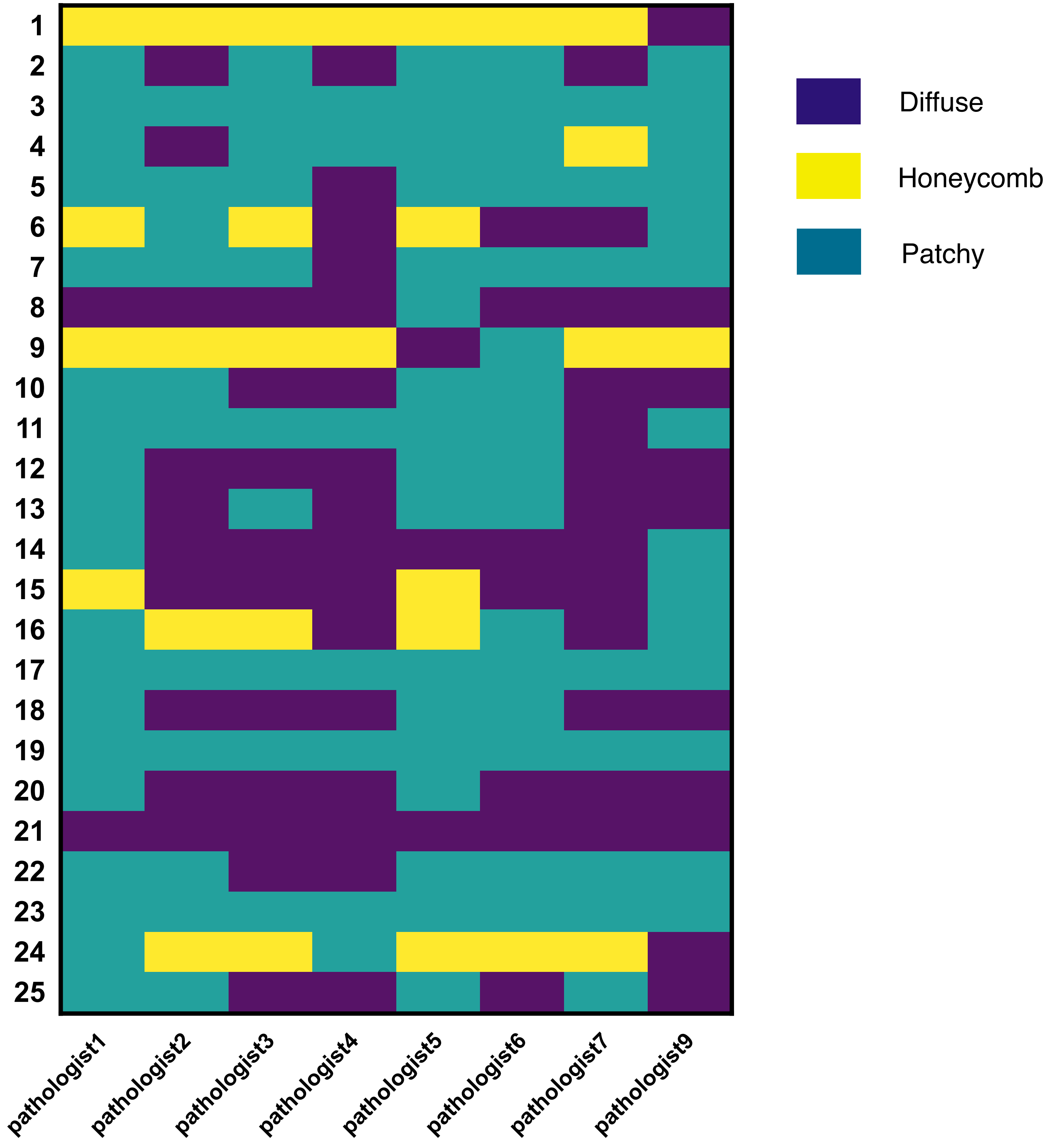

Supplemental figure 3b

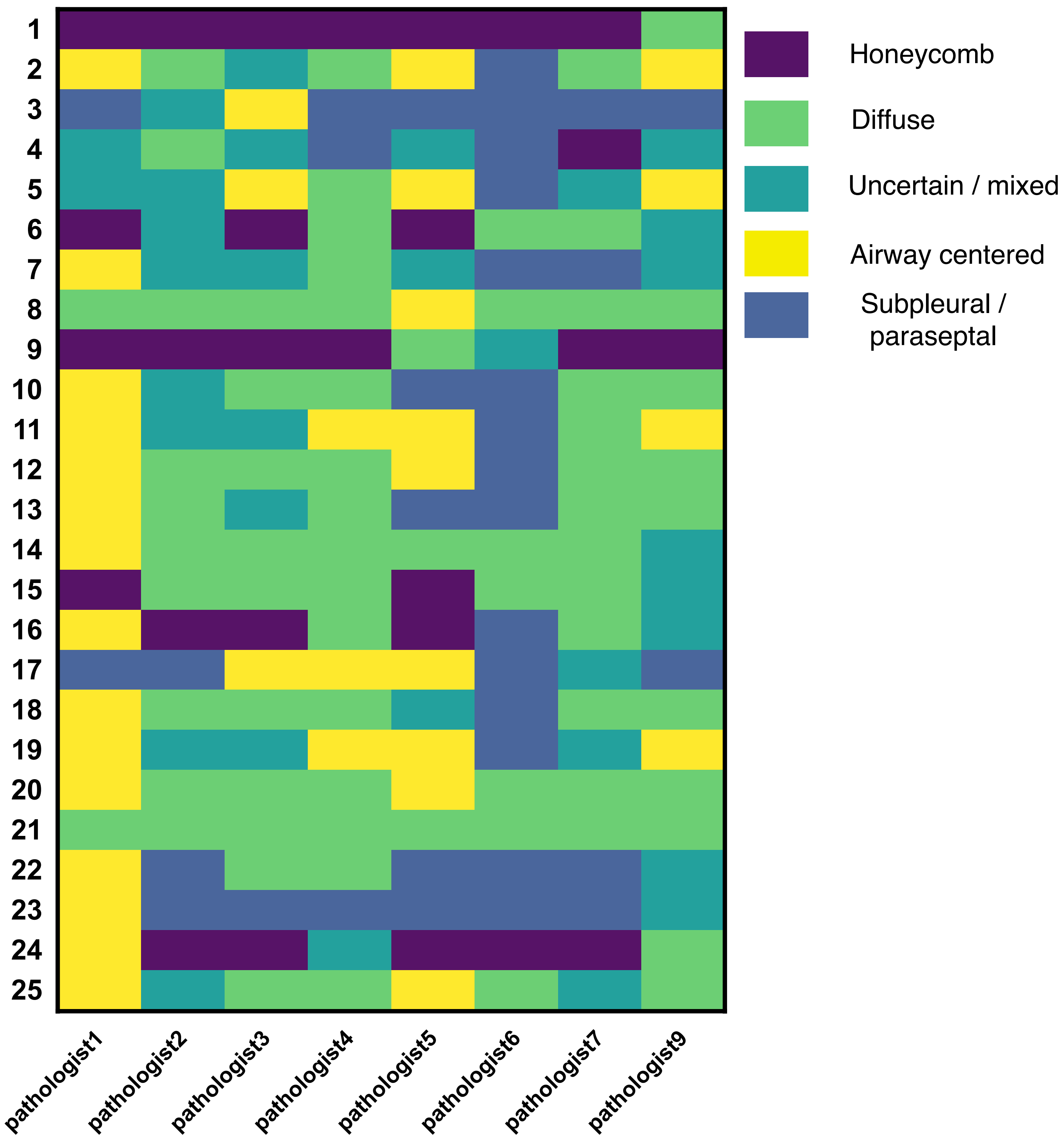

Supplemental figure 3c

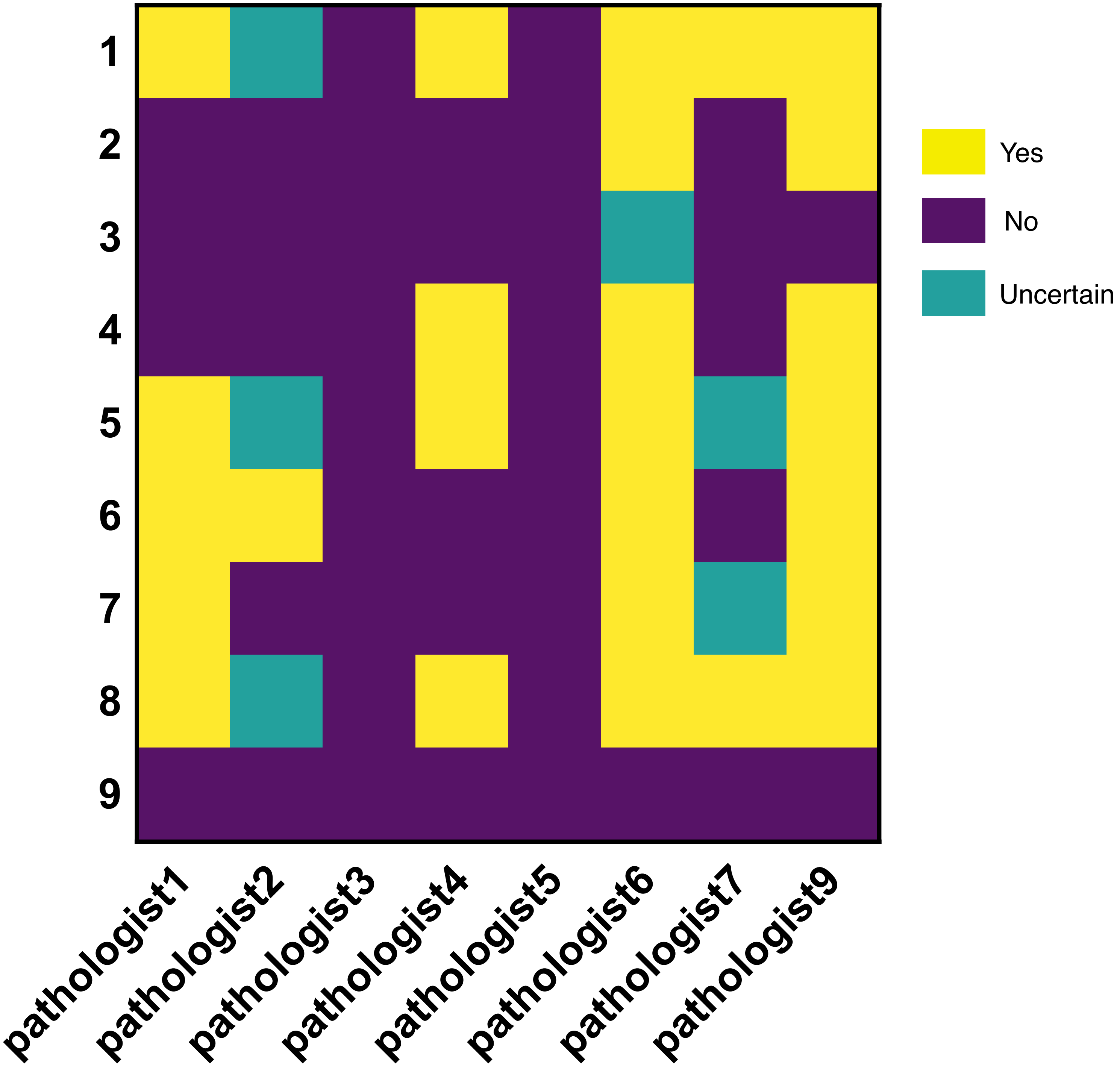

Supplemental figure 4

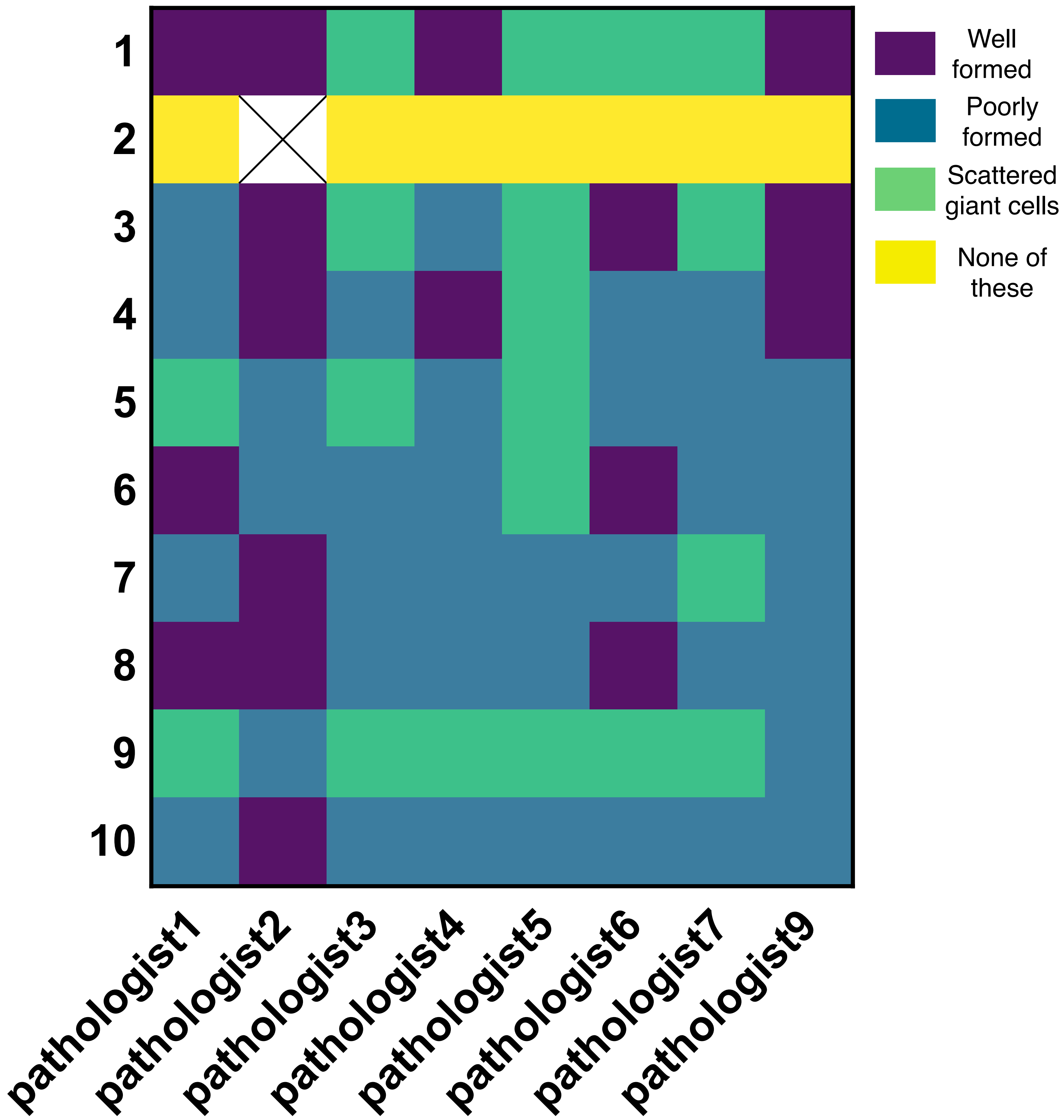

Supplemental figure 5
